# Suspected C5 Nerve Root Injury Temporally Associated With Stellate Ganglion Block for Refractory Insomnia: A Case Report

**DOI:** 10.1002/ccr3.73603

**Published:** 2026-09-29

**Authors:** Xiaochen Ji, Zhiqiang Niu, Yabo Wang

**Affiliations:** ^1^ Anaesthesia Medicine Center Cangzhou Central Hospital Cangzhou Hebei China

**Keywords:** C5 nerve root injury, case report, neurological complication, refractory insomnia, stellate ganglion block, ultrasound guidance

## Abstract

Suspected C5 nerve root injury temporally associated with stellate ganglion block is uncommon, and causality may be uncertain. Immediate radiating shoulder pain, lateral upper‐arm numbness, and deltoid weakness require prompt cessation, neurological assessment, electrophysiological confirmation, neurotrophic treatment, and rehabilitation. Careful needle visualization and transparent reporting of diagnostic uncertainty are essential.

## 1 Introduction

Stellate ganglion block (SGB) is a clinical technique that involves injecting local anesthetics into the stellate ganglion region to modulate autonomic nervous system activity. In recent years, the use of SGB has been explored in pain management and in selected patients with refractory insomnia, with potential benefits for symptom control and quality of life [1]. However, C5 nerve root injury or C5‐distribution neurological symptoms occurring after this procedure have not been frequently reported. Once it occurs, it may result in clinically significant motor and sensory dysfunction [2]. The clinical manifestations of C5 nerve root injury typically include radiating pain, decreased upper limb strength, and corresponding sensory deficits. These symptoms may affect patients' daily activities and functional recovery [3].

During SGB, deviations in needle trajectory, excessive puncture depth, or anatomical variations may theoretically increase the risk of unintended contact with adjacent neural structures [4]. Therefore, procedural safety, accurate needle‐tip visualization, and careful assessment of neurological symptoms during and after injection are important considerations in clinical practice [5]. Understanding the diagnostic features and potential mechanisms of C5 nerve root injury is essential for improving early recognition, timely management, and patient safety.

The present case describes a 49‐year‐old female patient who underwent SGB for refractory insomnia and immediately developed radiating pain in the right shoulder and weakness in the right upper limb after the procedure. Electromyography revealed findings consistent with acute C5 nerve root injury. Compared with previously reported neurological complications in other clinical contexts, this case occurred during SGB performed for sleep disorder treatment, suggesting that potential neurological risks should also be considered when SGB is used for nontraditional indications. This case may provide practical clinical reminders regarding ultrasound‐guided needle placement, careful monitoring of patient‐reported symptoms, and early neurological assessment after the procedure [6, 7].

In summary, although C5 nerve root injury following SGB has not been frequently reported, its potential clinical consequences should not be overlooked. Through this case analysis, we aim to emphasize the importance of procedural safety, early recognition of neurological complications, and standardized clinical practice during SGB.

## 2 Case Presentation/Examination

This case report was prepared in accordance with the CARE reporting guidelines. This case involved a 49‐year‐old female patient who presented with refractory insomnia lasting 6 months. Before SGB, her sleep status was evaluated using clinical history, medical records, and a structured sleep interview. She reported an average total sleep duration of approximately 4 h per night, a sleep latency of approximately 90 min, and frequent nocturnal awakenings 4–5 times per night. The Pittsburgh Sleep Quality Index (PSQI) score was 16, and the Insomnia Severity Index (ISI) score was 21, supporting a diagnosis of moderate‐to‐severe chronic insomnia. Previous treatments included sleep hygiene education, oral estazolam, and melatonin, with limited clinical improvement. The patient had a history of hypertension that was well controlled with medication. She denied a history of cervical spondylosis, trauma, or neurological disorders, and reported no obvious snoring, witnessed apnea, abnormal sleep‐related behaviors, restless legs, or periodic limb movements during sleep.

The patient mainly reported difficulty initiating and maintaining sleep, accompanied by daytime fatigue, irritability, and impaired concentration related to poor sleep quality. Polysomnography was not performed because she had no prominent clinical features suggestive of obstructive sleep apnea, parasomnia, or periodic limb movement disorder. Chronic insomnia was diagnosed based on her clinical symptoms, sleep history, sleep scale assessments, and the International Classification of Sleep Disorders, Third Edition (ICSD‐3). Neurological examination and cervical MRI showed no abnormalities. After clinical evaluation, right stellate ganglion block (SGB) was performed to modulate autonomic nervous system activity.

## 3 Methods

The procedure was performed by an experienced anesthesiologist trained in ultrasound‐guided stellate ganglion block. The patient was placed in the supine position with a thin pillow beneath the right shoulder, and the head was gently rotated to the left. After standard skin antisepsis and sterile draping, a high‐frequency 10–12 MHz linear‐array transducer covered with a sterile sheath was positioned transversely at the level of the cricoid cartilage to obtain a short‐axis view of the C6 vertebral level. The carotid artery, internal jugular vein, thyroid gland, trachea, anterior tubercle of the C6 transverse process, longus colli muscle, and prevertebral fascia were identified. Color Doppler imaging was used to exclude vascular structures along the planned needle trajectory.

A sterile 22‐gauge block needle was introduced from the lateral side using an in‐plane, lateral‐to‐medial approach. Under real‐time ultrasound guidance, the needle was advanced toward the prevertebral fascial plane on the anterior surface of the longus colli muscle while avoiding adjacent vessels and neural structures. After repeated negative aspiration for blood or cerebrospinal fluid, 3 mL of 0.2% ropivacaine combined with 1 mL of compound betamethasone was injected slowly and incrementally. The distribution of the injectate along the fascial plane and the patient's sensory symptoms were monitored throughout the procedure. No radiating pain, paresthesia, or motor symptoms were reported by the patient during needle advancement or injection, and the planned injection was therefore completed.

Importantly, definite intraneural or intrafascicular injection was not unequivocally identified during real‐time ultrasound monitoring. The stored ultrasound image demonstrated the needle trajectory toward the intended prevertebral region but, as a static image, did not provide sufficient evidence to confirm penetration of or injection into a specific neural structure (Figure 1). Therefore, the possibility of inadvertent needle–nerve contact or unintended perineural/intraneural spread was considered retrospectively after the subsequent neurological symptoms developed rather than being definitively recognized during the injection itself.

**FIGURE 1:**
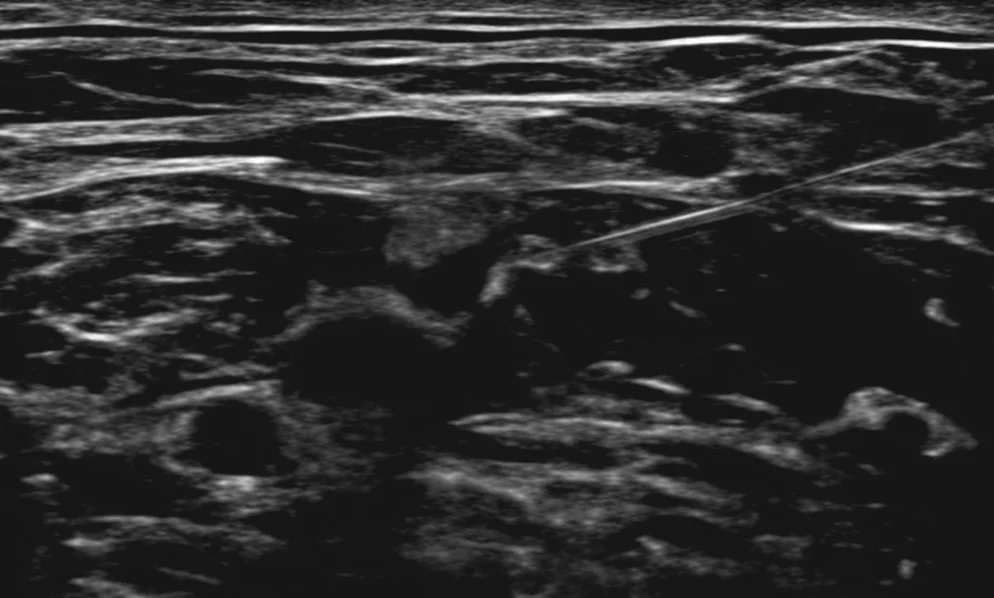
Ultrasound‐guided needle trajectory during stellate ganglion block.

Approximately 1 min after completion of the injection and withdrawal of the needle, the patient reported sudden severe electric shock‐like radiating pain extending from the right shoulder to the lateral aspect of the upper arm, followed by weakness of shoulder abduction and elbow flexion. Because the neurological symptoms occurred only after completion of the block, there was no opportunity for an intraprocedural cessation of injection in response to patient‐reported neurological symptoms. No additional injection, repeat needle placement, or further stellate ganglion block was subsequently performed. Immediate neurological assessment was initiated to determine the distribution and severity of the deficit.

Objective neurological examination included assessment of motor strength, cutaneous sensation, and deep tendon reflexes. Two hours after the procedure, physical examination showed Medical Research Council (MRC) grade 3/5 strength of the right deltoid and biceps brachii muscles and MRC grade 4/5 strength of the brachioradialis. Pinprick sensation was reduced over the lateral aspect of the right upper arm, corresponding predominantly to the C5 dermatome, and the right biceps tendon reflex was diminished compared with the contralateral side. These findings demonstrated a clinically concordant motor, sensory, and reflex abnormality predominantly involving the C5 distribution.

Emergency cervical MRI was performed to exclude cervical disc herniation, hematoma‐related compression, or another acute structural lesion responsible for the neurological deficit. No acute compressive lesion capable of explaining the sudden neurological symptoms was identified. A dedicated MRI of the right brachial plexus was subsequently recommended to further evaluate the C5 nerve root and upper trunk; however, the patient declined further imaging, and brachial plexus MRI was therefore not performed.

On post‐procedural day 3, electromyography was performed because the patient continued to have radiating shoulder pain, weakness of shoulder abduction and elbow flexion, and sensory disturbance in the C5 dermatome after the expected duration of local anesthetic effect had passed (Presented in Table 1). Electromyography showed abundant spontaneous potentials and reduced recruitment in the right deltoid and biceps brachii muscles. Because these muscles are predominantly innervated by the C5 nerve root, the abnormalities supported acute C5 motor root involvement. Sensory nerve action potentials were preserved, which favored a preganglionic nerve root lesion rather than an isolated distal peripheral nerve injury. However, the clinical distribution of deltoid and biceps weakness also overlaps with partial upper‐trunk brachial plexus involvement. Because dedicated brachial plexus MRI was not performed and repeat electromyography was not available, definitive anatomical localization between the C5 nerve root and the upper trunk of the brachial plexus could not be established. Therefore, the electrophysiological and clinical findings were interpreted as favoring C5‐predominant involvement rather than definitively confirming an isolated C5 nerve root lesion. Taken together with the abrupt postprocedural onset, the neurological examination, the absence of a corresponding compressive lesion on cervical MRI, and the electrophysiological findings, the overall pattern supported a suspected acute C5‐predominant neural injury temporally associated with SGB. However, because definite intraneural injection was not documented on ultrasound, the precise mechanism of injury could not be established solely from the procedural imaging.

**TABLE 1:** Needle electromyographic findings of the right deltoid supporting C5‐predominant involvement on post‐procedural day 3.

Parameter	Result
Muscle	Right deltoid
Insertional activity	1+
Fibrillation potentials	1+
Positive sharp waves	1+
Fasciculation potentials	−
Other spontaneous activity	−
Overall spontaneous activity	2+
Polyphasic potentials	+
MUP morphology	Essentially normal, with some polyphasic MUPs
MUP amplitude	1.2 mV
MUP duration	10.5 ms
Recruitment pattern	Discrete pattern with reduced recruitment
Recruitment during maximal voluntary contraction	Markedly reduced

Differential diagnoses included acute compressive cervical radiculopathy, shoulder or rotator cuff pathology, isolated axillary or musculocutaneous neuropathy, and upper‐trunk brachial plexopathy. The absence of an acute compressive lesion on cervical MRI and the combined motor, sensory, reflex, and electrophysiological findings made acute compressive radiculopathy and isolated distal peripheral nerve injury less likely. However, partial upper‐trunk brachial plexus involvement could not be completely excluded because dedicated brachial plexus MRI and repeat electromyography were unavailable.

After a procedure‐associated C5‐predominant neurological injury was suspected, treatment was initiated with mecobalamin and vitamin B1 for neurotrophic support, combined with physical therapy, including ultrashort‐wave therapy and medium‐frequency electrical stimulation.

## 4 Conclusion and Results

After 2 weeks of treatment, the patient reported marked pain relief, and muscle strength showed gradual improvement. At the 3‐month follow‐up, MRC strength of the right deltoid and biceps brachii had recovered to 4+/5, sensory abnormalities had largely resolved, and insomnia symptoms had also improved.

Pain intensity, assessed using a 0–10 visual analogue scale, decreased from 8 at symptom onset to 3 at the 2‐week follow‐up and further to 1 at the 3‐month follow‐up, indicating sustained symptomatic improvement. In addition to VAS assessment, objective neurological follow‐up included repeat manual muscle testing using the Medical Research Council (MRC) scale and clinical sensory examination. At the 3‐month follow‐up, MRC strength of the right deltoid and biceps brachii had improved from 3/5 to 4+/5, accompanied by substantial improvement of the C5‐distribution sensory deficit. No repeat electromyography or validated upper‐extremity functional outcome scale was performed during follow‐up.

In this case, a C5‐predominant neurological deficit developed within minutes after completion of SGB, providing a strong temporal relationship between the procedure and the neurological event. The patient had no corresponding neurological deficit before SGB, whereas postprocedural examination demonstrated concordant abnormalities in motor strength, C5‐distribution sensation, and the biceps tendon reflex. Together with the subsequent electrophysiological findings, these observations increased the likelihood of a procedure‐associated neural injury, although they did not establish a specific mechanism such as confirmed intraneural injection.

Although C5 nerve root injury or C5‐distribution neurological symptoms after SGB have not been frequently reported, they may result in clinically relevant motor and sensory dysfunction. The present case suggests that neurological complications may become clinically apparent only after completion of the block, even when the patient reports no radiating pain or paresthesia during needle advancement or injection. Clinicians should therefore monitor neurological status not only during injection but also during the immediate postprocedural period. Serial assessment of muscle strength, sensory function, and reflexes may be useful for monitoring neurological recovery. New radiating pain, focal weakness, sensory loss, or reflex asymmetry should prompt timely neurological examination and appropriate imaging and electrophysiological evaluation. Careful needle advancement, continuous visualization of the needle tip, conservative injection volumes, and vigilant postprocedural assessment may help improve procedural safety.

## 5 Discussion

This case report describes suspected C5 nerve root injury temporally associated with ultrasound‐guided stellate ganglion block (SGB). Although neurological complications after SGB are not frequently reported, they may result in clinically significant sensory and motor dysfunction. Previous studies have described C5 and C6 nerve root injuries in other clinical contexts, including nerve root injection and cervical spine surgery [8, 9, 10]. In contrast, the present event occurred during SGB performed for refractory insomnia, a setting in which C5‐predominant neurological injury has rarely been described [11].

The possible mechanisms underlying C5‐distribution neurological symptoms after SGB may be multifactorial and may involve regional anatomical relationships, needle trajectory, injection depth, and the distribution of the injected solution [12]. Neurophysiological examination may be useful for confirming the diagnosis and assessing the extent of nerve root involvement when postoperative neurological deficits are suspected [5, 13, 14, 15].

In interpreting this neurological event, however, the procedural findings warranted caution. The patient reported no radiating pain, paresthesia, or weakness during needle advancement or injection, and real‐time ultrasound did not provide unequivocal evidence of intraneural or intrafascicular injection; therefore, a confirmed direct needle‐related or intraneural injection injury could not be established. Nevertheless, the onset of symptoms approximately 1 min after completion of SGB, together with newly developed and anatomically concordant C5‐predominant motor, sensory, and reflex abnormalities, strengthened the temporal and anatomical association between the procedure and the neurological event. Inadvertent needle–neural contact, perineural or limited intraneural spread, and injection‐related neural irritation therefore remained plausible mechanisms, although none could be definitively demonstrated. Accordingly, we interpreted the event as a suspected C5‐predominant neural injury temporally associated with SGB rather than a confirmed intraneural injection‐related C5 nerve root injury.

Previous magnetic resonance imaging evidence has shown that small‐volume SGB injectate may distribute from approximately the C5 to T1 vertebral levels; however, differences in the injection approach and target plane mean that this evidence supports only anatomical plausibility and does not demonstrate that comparable spread occurred in this patient [16]. Compound betamethasone was added to ropivacaine according to local clinical practice as a corticosteroid adjuvant, with the intention of attenuating local inflammatory responses and prolonging the therapeutic effect of the block. This rationale was extrapolated from studies in other regional anesthesia settings showing that betamethasone combined with ropivacaine may improve early analgesia and reduce inflammatory responses; however, direct evidence supporting betamethasone as an adjunct to SGB for refractory insomnia remains limited, and its specific contribution to the neurological event in this case could not be determined [17, 18]. Persistent weakness beyond the expected transient effect of local anesthetic, together with the subsequent electrophysiological abnormalities, made transient anesthetic spread alone less likely.

Electromyography was performed on post‐procedural day 3 because the neurological symptoms persisted beyond the expected transient effect of local anesthetic spread. It demonstrated abundant spontaneous potentials and reduced recruitment in the right deltoid and biceps brachii muscles, while sensory nerve action potentials were preserved. This pattern supported a preganglionic C5 nerve root lesion rather than an isolated distal peripheral nerve injury or typical postganglionic brachial plexus lesion. Nevertheless, because the EMG was performed at an early stage and neither repeat electromyography nor dedicated brachial plexus MRI was available, the evolution of denervation and partial upper‐trunk brachial plexus involvement could not be completely assessed. The early neurological assessment and electrophysiological evaluation therefore supported the diagnosis of acute C5 nerve root involvement and guided subsequent management [19].

Optimization of procedural techniques is important for reducing the risk of neurological symptoms or nerve irritation during SGB. Accurate ultrasound visualization of the needle tip, careful selection of the puncture route, avoidance of excessive puncture depth, and low‐volume injection may help reduce unintended spread toward adjacent neural structures. For example, compared with the traditional posterior tubercle approach, the anterolateral cervical approach may help reduce the risk of nerve root involvement in selected cases [20]. In addition, combining ultrasound guidance with low‐volume drug injection may limit diffusion to nearby nerve roots and improve procedural safety [21]. Standardized training and adherence to procedural protocols are also important for improving the safety of SGB [22]. Before SGB is performed, clinicians should evaluate the indication carefully and provide sufficient information regarding potential benefits, limitations, and neurological risks to support informed decision‐making [23].

This case has several limitations. As a single case report, the findings cannot be generalized to all patients undergoing SGB. Individual anatomical variations, comorbidities, puncture conditions, and injection characteristics may influence the risk of neurological complications. Moreover, because repeat electromyography was not obtained, the evolution of denervation and recovery could not be objectively characterized, and definitive anatomical localization between the C5 nerve root and the upper trunk of the brachial plexus remained uncertain. Although neurological follow‐up included repeat MRC muscle strength assessment and clinical sensory examination, repeat electromyography and validated upper‐extremity functional outcome scales were not obtained. This limited objective assessment of electrophysiological recovery and functional improvement over time. Accordingly, the diagnosis should be interpreted as probable C5‐predominant involvement rather than definitive isolated C5 nerve root injury. Further studies are needed to clarify possible mechanisms, risk factors, and preventive strategies for neurological symptoms temporally associated with SGB.

## Author Contributions

**Xiaochen Ji:** funding acquisition, project administration, software, writing – original draft. **Zhiqiang Niu:** funding acquisition, software. **Yabo Wang:** funding acquisition.

## Funding

This study was supported by the 2025 Hebei Province Medical Science Research Project Plan (No. 20251530), “Study on the Advantages of Ultrasound‐Guided Stellate Ganglion Block Combined with Propofol in the Treatment of Menopausal Insomnia”.

## Ethics Statement

This study was approved by the Ethics Committee of Cangzhou Central Hospital (Approval No. 2024‐332‐03) and conducted in accordance with the Declaration of Helsinki. Written informed consent for publication of this case report and accompanying clinical information was obtained from the patient.

## Consent

Written informed consent was obtained from the patient for publication of this case report and any accompanying images or clinical information.

## Conflicts of Interest

The authors declare no conflicts of interest.

## Data Availability

The data that support the findings of this case report are available from the corresponding author upon reasonable request. The data are not publicly available due to privacy or ethical restrictions.
